# Prophages in Lactobacillus reuteri Are Associated with Fitness Trade-Offs but Can Increase Competitiveness in the Gut Ecosystem

**DOI:** 10.1128/AEM.01922-19

**Published:** 2019-12-13

**Authors:** Jee-Hwan Oh, Xiaoxi B. Lin, Shenwei Zhang, Stephanie L. Tollenaar, Mustafa Özçam, Case Dunphy, Jens Walter, Jan-Peter van Pijkeren

**Affiliations:** a Department of Food Science, University of Wisconsin—Madison, Madison, Wisconsin, USA; b Department of Agriculture, Food and Nutritional Science, University of Alberta, Edmonton, Canada; c Department of Biological Sciences, University of Alberta, Edmonton, Canada; University of Tartu

**Keywords:** *Lactobacillus reuteri*, bacteriophages, intestinal colonization, lysogen, microbial ecology, probiotics, prophage

## Abstract

The gut microbiota harbors a diverse phage population that is largely derived from lysogens, which are bacteria that contain dormant phages in their genome. While the diversity of phages in gut ecosystems is getting increasingly well characterized, knowledge is limited on how phages contribute to the evolution and ecology of their host bacteria. Here, we show that biologically active prophages are widely distributed in phylogenetically diverse strains of the gut symbiont Lactobacillus reuteri. Nearly all human- and rodent-derived strains, but less than half of the tested strains of porcine origin, contain active prophages, suggesting different roles of phages in the evolution of host-specific lineages. To gain insight into the ecological role of L. reuteri phages, we developed L. reuteri strain 6475 as a model to study its phages. After administration to mice, L. reuteri 6475 produces active phages throughout the intestinal tract, with the highest number detected in the distal colon. Inactivation of *recA* abolished *in vivo* phage production, which suggests that activation of the SOS response drives phage production in the gut. In conventional mice, phage production reduces bacterial fitness as fewer wild-type bacteria survive gut transit compared to the mutant lacking prophages. However, in gnotobiotic mice, phage production provides L. reuteri with a competitive advantage over a sensitive host. Collectively, we uncovered that the presence of prophages, although associated with a fitness trade-off, can be advantageous for a gut symbiont by killing a competitor strain in its intestinal niche.

**IMPORTANCE** Bacteriophages derived from lysogens are abundant in gut microbiomes. Currently, mechanistic knowledge is lacking on the ecological ramifications of prophage carriage yet is essential to explain the abundance of lysogens in the gut. An extensive screen of the bacterial gut symbiont Lactobacillus reuteri revealed that biologically active prophages are widely distributed in this species. L. reuteri 6475 produces phages throughout the mouse intestinal tract, but phage production is associated with reduced fitness of the lysogen. However, phage production provides a competitive advantage in direct competition with a nonlysogenic strain of L. reuteri that is sensitive to these phages. This combination of increased competition with a fitness trade-off provides a potential explanation for the domination of lysogens in gut ecosystem and how lysogens can coexist with sensitive hosts.

## Abstract (precis)

Bacteriophages derived from lysogens are abundant in gut microbiomes. Currently, mechanistic knowledge is lacking on the ecological ramifications of prophage carriage yet is essential to explain the abundance of lysogens in the gut. An extensive screen of the bacterial gut symbiont Lactobacillus reuteri revealed that biologically active prophages are widely distributed in this species. L. reuteri 6475 produces phages throughout the mouse intestinal tract, but phage production is associated with reduced fitness of the lysogen. However, phage production provides a competitive advantage in direct competition with a nonlysogenic strain of L. reuteri that is sensitive to these phages. This combination of increased competition with a fitness trade-off provides a potential explanation for the domination of lysogens in gut ecosystem and how lysogens can coexist with sensitive hosts.

## INTRODUCTION

The intestinal tract of vertebrates is dominated by bacteria and their viruses—bacteriophages (1–3), here called phages. Phages may follow one of two life cycles: lytic or lysogenic. Lytic phages infect their host, replicate inside the host cell, and subsequently lyse the bacterium to release their progeny. Experiments in laboratory communities revealed that lytic phages and their host are in a continuous arms race that leads to selection of bacteria that have developed resistance, which in turn leads to selection for phages that have adapted (4,5). This indicates that sensitive and resistant bacterial strains can coexist both with the lytic phage and each other over long periods due to the fitness cost of resistance, especially when resources are limited (6). These interactions provide explanations for the vast diversity of lytic phages in microbial ecosystems and their dynamics observed in nature.

Much less is known about the ecology and evolution of lysogenic phages, especially in gut ecosystems. In human feces, it is estimated there are ∼10^11^ bacterial cells g^−1^ and up to 10^10^ virus-like particles g^−1^ (7, 8), which include temperate phages (3). Temperate phages infect their host and integrate in the bacterial chromosome, which could lead to a long-lasting relationship (9). However, lysogeny can come with a fitness cost. First, the cell needs to maintain additional genetic material (10–12), which can be as much as 16% of the total genome content (9). Second, activation of lysogenic phages can kill the host (13–15). Despite the fitness burden of prophage carriage, in gut ecosystems half of the identified virus-like particles are derived from lysogens (16–18). Yet, evidence exists that development of phage resistance comes at a fitness cost (19), which is amplified in less productive environments (20). On the other hand, while prophages are dormant, they can provide protection against infection by similar phages (21) and are a source of genetic variation that has been proposed to provide an advantage during the evolution of bacterial species (22–26). Also, prophage carriage has been associated with increased competitiveness. *In vitro* competition experiments between lysogenic and phage-sensitive Salmonella enterica serovar Typhimurium strains revealed that prophages provide a competitive advantage (25). However, so far only two ecological studies with intestinal bacteria and their prophages have been conducted in the gut environment. In one study, competition experiments in the mouse gut between a nonlysogen and lysogen strain of Enterococcus faecalis revealed that the lysogen strain had outcompeted the nonlysogen by 1.5-fold (27); however, the experiment was limited to 24 h. A more recent study with Escherichia coli and phage λ demonstrated that phage carriage comes at a fitness cost but yields a competitive advantage when in direct competition with E. coli bacteria sensitive to phage λ (14).

The available information points to both beneficial and detrimental effects of prophages in gut microbes. However, it is still unclear to what extent prophages of a mutualistic host-associated microbe impact gut fitness. Currently, experimental evidence is lacking to substantiate claims linking beneficial or detrimental effects to the presence of prophages. This lack of knowledge stems from the absence of mechanistic studies that investigate to what extent prophages impact gut bacterial ecology and fitness in realistic model organisms in relevant experimental settings.

Lactobacillus reuteri is a Gram-positive bacterial gut symbiont that can be found in the intestinal tract of vertebrates, including pigs, mice, rats, birds, and humans (28), and has been established as a model to study the evolution of gut symbionts with their hosts (29). Comparative genome analyses combined with functional experiments in animals revealed host-adapted phylogenetic lineages whose genome content reflects niche characteristics in the respective host species (30–32). In addition, three genome editing tools have been developed for the human isolate L. reuteri 6475, including single-stranded DNA recombineering (33), CRISPR-Cas genome editing (34), and a counterselection marker (35). We applied these tools to develop L. reuteri 6475 as a model to study the functions of prophages. The strain contains two biologically active prophages, LRΦ1 and LRΦ2, which are members of the *Siphoviridae* family. Each prophage genome is 43 kb and is induced in the gastrointestinal tract (15). Exposure to the short-chain fatty acids acetic acid, propionic acid, and/or butyric acid or metabolism of fructose activates the Ack pathway, which, in turn, activates prophages in a RecA-dependent manner. These findings were not species or strain specific as exposure to short-chain fatty acids also promoted phage production in Lactococcus lactis and in L. reuteri ATCC 55730 (15), a strain that is genetically distinct from L. reuteri 6475 (36). While our previous study unraveled mechanisms by which prophages are induced in gut ecosystems, they have not revealed how prophages evolve with L. reuteri and how they influence its ecology in the gut.

In this study, we show that active prophages are distributed among a broad range of phylogenetically diverse strains within the species Lactobacillus reuteri, which suggests that the gut environment selects for a temperate lifestyle. Using L. reuteri 6475 and its isogenic prophage deletion derivatives, we determined the spatial scale of phage production in the gut and the mechanisms of induction. Also, we established the role of prophages for ecological fitness both in the presence of a complex microbiota and in direct competition with a sensitive strain.

## RESULTS

### Active prophages are broadly represented in strains of Lactobacillus reuteri.

**FIG 1:**
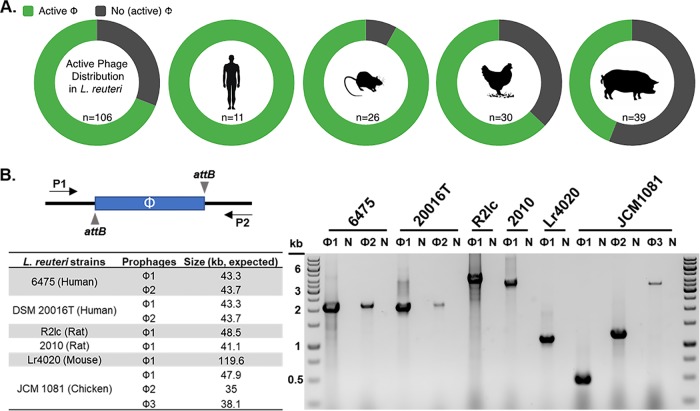
Distribution of active prophages in L. reuteri. (A) The distribution of active (green) and nonactive (gray) prophages as determined by mitomycin C induction in L. reuteri strains with different host origins. “n” represents the number of strains tested in total (*n* = 106) or per origin. (B) Schematic of the experimental design to identify prophage excision showing oligonucleotides (P1 and P2) flanking the prophage (Φ) and the *attB* sites. The table shows select L. reuteri strains with their predicted prophage(s) (see the text for details). The agarose gel shows PCR amplicons using oligonucleotides flanking the predicted prophage(s). Strains and their prophages are listed above the gel. “N” indicates the negative (water) control.

To gain insight into the role of prophages in the evolution of L. reuteri, we first determined the presence of active phages in strains that cover the known phylogenetic diversity of the species. We mapped the prophage distribution in 28 L. reuteri strains of vertebrate origin and identified 17 genomes that are predicted to encode intact prophages, while nearly all genomes contained incomplete prophages or phage remnants (Table 1). To expand on this observation, we assessed the distribution of biologically active prophages in this species by screening an extensive library of L. reuteri strains with mitomycin C (MMC), which activates the bacterial SOS response and cues the prophages to excise and lyse the bacterial cell. We tested 106 strains, including 14 strains for which a genome sequence is available (Table 1; see Fig. S2 in the supplemental material), that originate from the different phylogenetic lineages within the species (32), and are derived from different host origins: humans (*n* = 11), pigs (*n* = 39), chickens (*n* = 30), and rodents (*n* = 26). Overall, 73 out of 106 strains (69%) were induced by MMC (Fig. 1A; Fig S2). When we analyzed the distribution of active prophages by host origin, we observed that human and rodent isolates have a high distribution of active prophages (100% and 88%, respectively), while fewer active prophages were identified in chicken and pig isolates (63% and 44%, respectively) (Fig. 1A).

**TABLE 1:** *In silico* analysis and mitomycin C induction of prophages present in L. reuteri genomes

Strain	Genome accession no.	Origin	Status	Size (bp)	No. of prophages				MMC induction
					Intact	Questionable	Incomplete	Total	
JCM 1112	AP007281.1	Human	Complete	2,039,414	2	0	2	4	Yes
DSM 20016	CP000705.1	Human	Complete	1,999,618	3	1	4	8	Yes
SD2112	CP002844.1	Human	Complete	2,264,399	3	1	7	11	Yes
IRT	NZ_CP011024.1	Human	Complete	1,993,967	1	0	2	3	ND^*b*^
121	GCA_001889975.1	Human	Complete	2,302,234	0	1	3	4	ND
ATCC PTA 6475	ACGX00000000.2	Human	Scaffold	2,067,914	2	1	1	4	Yes
CF48-3A	ACHG00000000.1	Human	Scaffold	2,107,903	2	1	1	4	ND
ATCC PTA-4659	ACLB00000000.1	Human	Scaffold	2,015,721	3	0	2	5	Yes
MM34-4A	GCA_002112805.1	Human	Scaffold	2,152,944	0	0	0	0	ND
M27U15	GCA_002112195.1	Human	Scaffold	2,035,662	0	2	0	2	ND
I49	NZ_CP015408.2	Mouse	Complete	2,063,604	0	1	6	7	ND
mlc3	AEAW00000000.1	Mouse	Scaffold	2,018,630	1	0	1	2	Yes
lpuph	AEAX00000000.1	Mouse	Scaffold	2,116,621	1	0	4	5	Yes
LR0	GCA_002156605.1	Mouse	Contig	2,148,567	0	0	2	2	ND
TD1	NC_021872.1	Rat	Complete	2,145,445	1	1	2	4	ND
100-23	AAPZ00000000.2	Rat	Scaffold	2,305,557	3	0	5	8	Yes
ATCC 53608	CACS00000000.2	Pig	Complete	2,091,243	0	0	1	1	No
I5007	CP006011.1	Pig	Complete	1,947,706	1	0	2	3	No
pg-3b	2599185334 (IMG)^*a*^	Pig	Scaffold	1,890,545	0	0	0	0	ND
ZLR003	NZ_CP014786.1	Pig	Complete	2,234,097	1	2	1	4	ND
20-2	2599185332 (IMG)	Pig	Scaffold	2,232,947	0	0	0	0	ND
lp167-67	2599185361 (IMG)	Pig	Scaffold	2,015,596	1	0	0	1	No
3c6	2599185333 (IMG)	Pig	Scaffold	1,934,800	0	0	1	1	No
P43	GCA_001705505.1	Pig	Contig	2,151,063	2	0	4	6	ND
JCM 1081	GCA_002112225.1	Chicken	Scaffold	2,313,528	2	1	3	6	Yes
1366	GCA_002112185.1	Chicken	Scaffold	2,062,915	0	0	3	3	No
CSF8	GCA_002112245.1	Chicken	Scaffold	1,952,049	0	0	1	1	Yes
An71	GCA_002159305.1	Chicken	Contig	2,280,851	1	0	2	3	ND

a IMG, Integrated Microbial Genomes database by Joint Genome Institute (JGI).

b ND, not determined.

Since mitomycin C may be toxic to certain strains at certain concentrations, we performed PCR with oligonucleotides flanking the prophage genome to predict excision upon mitomycin C treatment. From the pool of 106 strains, we selected 16 strains (indicated with an asterisk in Fig. S2) that lysed upon mitomycin C induction and for which a genome sequence is available. Using the online tool PHASTER we identified 21 presumptive intact prophage genomes distributed over these 16 strains. Eleven putative prophage genomes were located in a single contig, which did not allow us to pinpoint the integration site in the bacterial genome. For the remaining 10 prophage genomes, distributed over six strains, we designed oligonucleotides flanking the predicted prophage integration site. Each strain was exposed to mitomycin C, and PCR analysis yielded amplicons in all reactions (Fig. 1B), suggesting prophage excision. Collectively, our analyses demonstrate that biologically active prophages are widely distributed in L. reuteri.

### Construction of an experimental system to study the effect of L. reuteri 6475 prophages on their host.

**FIG 2:**
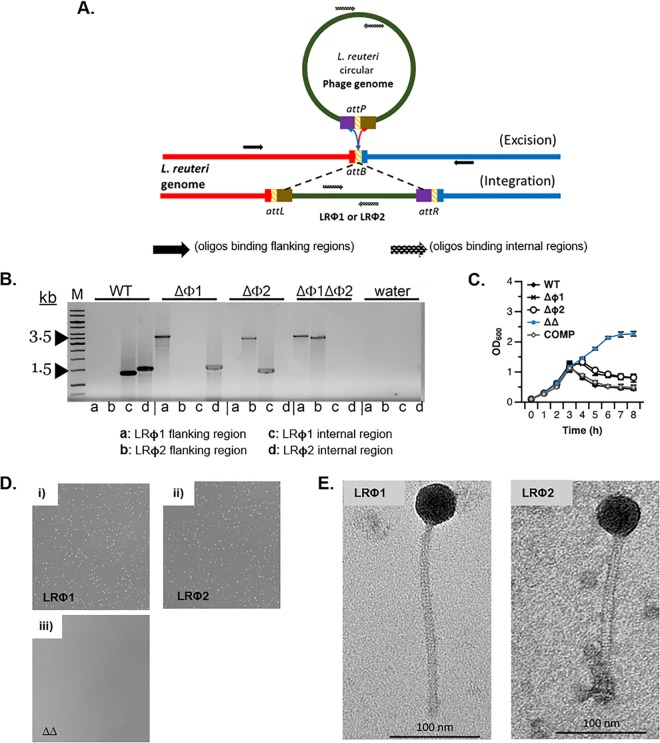
Development of a model system to study prophages in L. reuteri 6475. (A) Location of oligonucleotides on the prophage and bacterial chromosome to screen for integrated or deleted prophages. (B) PCR amplification to confirm prophage deletion(s). Lane M is the molecular size marker lane. (C) Growth curves following mitomycin C induction of L. reuteri wild-type (WT), the isogenic mutants lacking prophage 1 (ΔΦ1), prophage 2 (ΔΦ2), or both prophages (ΔΔ), and a derivative of the ΔΔ mutant in which both prophages were restored (COMP). (D) Plaque assay with supernatants derived from mitomycin C-treated cultures of the L. reuteri ΔΦ2 (i), ΔΦ1 (ii), and ΔΦ1 ΔΦ2 (iii) mutants which contain LRΦ1, LRΦ2, or no phage, respectively. (E) Transmission electron micrographs of the LRΦ1 (left) and LRΦ2 (right) particles.

Recently, we developed a derivative of L. reuteri 6475, a human breast milk isolate (32), lacking both intact prophage genomes to yield the L. reuteri ΔΦ1 ΔΦ2 double mutant strain, and we developed a sensitive host to enumerate phage production by L. reuteri 6475 (15). The sensitive host is a derivative of the L. reuteri ΔΦ1 ΔΦ2 strain, which lacks the corresponding *attB* sites in the genome. Here, we expanded the platform by construction of single prophage deletions to yield the L. reuteri ΔΦ1 and L. reuteri ΔΦ2 single mutant strains (see Materials and Methods for details). The corresponding genotypes were confirmed by PCR (Fig. 2A and B). Whole-genome sequencing of the phage deletion derivatives revealed that the L. reuteri ΔΦ1 ΔΦ2 strain acquired four single nucleotide polymorphisms (SNPs), one insertion, and one deletion mutation (Table 2). To link potential phenotypical differences with deletion of prophage(s) rather than the acquired SNPs, we generated a derivative of the L. reuteri ΔΦ1 ΔΦ2 mutant in which we restored each prophage in the chromosome, which here we refer to as the prophage-complemented strain (COMP). We confirmed by Sanger sequencing that the complemented version is a derivative of the L. reuteri ΔΦ1 ΔΦ2 strain as we identified the same SNPs in both the L. reuteri ΔΦ1 ΔΦ2 mutant and the complemented strain (data not shown).

**TABLE 2:** Mutations identified following whole-genome sequencing in L. reuteri-derivatives used in this study

Gene ID (JCM1112)	Annotation	DNA sequence change (amino acid sequence change)^*a*^			
		ΔФ1	ΔФ2	SH	ΔФ1 ΔФ2
LAR_0044	FmtB (Mrp) protein	-	-	-	1268–1270delATA; 3-bp deletion
LAR_0099	ParB, chromosome partitioning	-	-	268G→T (A90S)	268G→T (A90S)
LAR_0011	DNA-binding response regulator	-	-	-	371C→T (T124I)
LAR_1262	GentR family transcription regulator	298G→A (V100I)	-	298G→A (V100I)	298G→A (V100I)
LAR_1266	IS*30* family transposon	257G→A (S86N)	-	257G→A (S86N)	257G→A (S86N)
LAR_1268	Dextransucrase protein	-	-	-	107_108insC; frameshift mutation

a -, identical to the wild-type sequence; ΔΦ1, L. reuteri 6475 mutant in which prophage 1 is deleted; ΔΦ2, L. reuteri 6475 mutant in which prophage 2 is deleted; SH, sensitive host lacking both prophages and their *attB* sites; ΔΦ1 ΔΦ2, derivative of SH in which *attB* sites were restored.

Next, we tested the dynamics of lysis of the L. reuteri wild-type (WT) strain and its derivatives following induction with MMC. We observed that the L. reuteri wild type lysed most efficiently upon MMC treatment; lysis was delayed in each of the single-prophage-deletion strains (Fig. 2C). The strain in which we restored both prophages showed a lysis pattern very similar to that of the wild type, while the L. reuteri ΔΦ1 ΔΦ2 double mutant continued to grow following exposure to MMC. The latter indicates that LRΦ1 and LRΦ2 are the only MMC-inducible prophages in the L. reuteri 6475 genome. Although the dynamics of lysis are different between the single-prophage-deletion strains and the strains harboring both prophages, the growth rates were comparable between strains (see Fig. S3A in the supplemental material).

The supernatants derived from MMC-induced cultures of the L. reuteri ΔΦ1 and ΔΦ2 single mutant strains, containing LRΦ2 and LRΦ1, respectively, were exposed to the sensitive host strain. Each phage yielded plaques (Fig. 2D), which shows that each phage can be detected and quantified. Through these single-prophage-deletion variants, the individual phage particles can be produced following MMC treatment, allowing their characterization. Transmission electron microscopy (TEM) analysis revealed that phage particles derived from Φ1 and Φ2 have a structure typical for *Siphoviridae* (Fig. 2E). The phage particles share a similar morphology, with the only noticeable difference that LRΦ1 has a slightly longer tail compared to LRΦ2. In contrast to previous findings (37), where a linear correlation was found between the amino acid length of the tail tape measure protein and the tail length of the corresponding virus particle, we found that the annotated tail tape measure proteins of both L. reuteri 6475 phages were similar in size (data not shown). Collectively, our analysis showed that L. reuteri 6475 has two biologically active phages, and we developed a model system to study and quantify each of the prophages.

### Prophages reduce fitness of L. reuteri 6475 during gastrointestinal transit.

**FIG 3:**
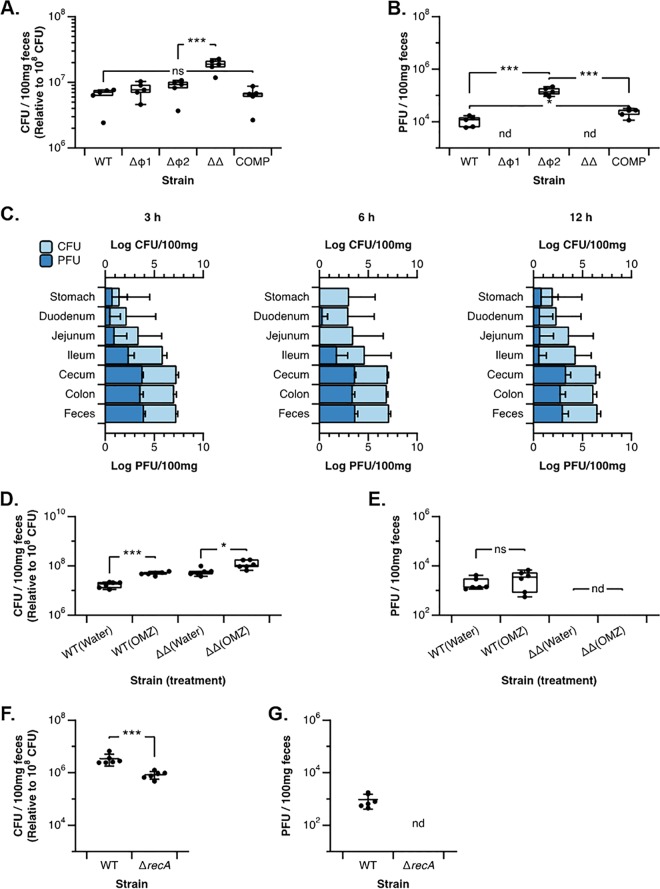
Characterization of L. reuteri 6475 prophages during gastrointestinal transit. (A) Cell numbers of the L. reuteri wild-type strain (WT), the L. reuteri ΔΦ1, ΔΦ2, and ΔΦ1 ΔΦ2 (ΔΔ) mutant strains, and the L. reuteriΔΦ1 ΔΦ2∷Φ1∷Φ2 complemented strain (COMP) as determined by bacterial culture from feces. ns, not significant; ***, *P* < 0.001. (B) Phage numbers expressed as PFU derived from mice administered the WT, ΔΦ1, ΔΦ2, ΔΔ, and COMP strains. *n* = 5 animals per group. nd, not detected; ***, *P* < 0.001. (C) Temporal and spatial analyses of the cell numbers of L. reuteri 6475 wild-type (light blue bars, top axis) and PFU (dark blue bars, bottom axis) normalized to 100 mg tissue/feces following administration of 10^8^ CFU at *t* = 0 h. Per time point, five animals were sacrificed. (D and E) Bacterial (D) and phage (E) counts following administration of L. reuteri strain 6475 (WT) or the ΔΦ1 ΔΦ2 mutant (ΔΔ) to mice that received regular drinking water (Water) or drinking water supplemented with omeprazole (OMZ). *n* = 6 animals per treatment group. (F and G) Bacterial (F) and phage (G) counts following administration of L. reuteri 6475 (WT) or 6475 Δ*recA* (*ΔrecA*). For all panels, CFU data are expressed per 100 mg feces and normalized to 10^8^ administered CFU and PFU data are expressed per 100 mg feces. ns, not significant; nd, not detected. *, *P* < 0.05; ***, *P* < 0.001 (*t* test analyses).

We previously demonstrated that both prophages reduce the fitness of L. reuteri following gastrointestinal (GI) transit (15); however, the impact of the individual prophages on gastrointestinal survival of L. reuteri was unknown. Therefore, we compared the fitness of the L. reuteri wild type to the single-prophage-deletion strains and the prophage-complemented strain. We administered conventional mice with 10^9^ bacteria for two consecutive days, followed by plate count analysis 24 h following the last gavage. L. reuteri 6475 does not colonize conventional mice but the strain can be detected up to 5 days in fecal samples (data not shown). In our experimental setup, we are thus measuring the impact that prophages have on the fitness of L. reuteri 6475 following gastrointestinal transit. As shown in Fig. 3A, we recovered a similar bacterial load of wild-type and COMP strains (6.19 × 10^6^ versus 6.18 × 10^6^ CFU/100 mg for WT versus COMP, respectively; *P = *0.99). Inactivation of single prophages only led to minor and nonsignificant 1.2- and 1.4-fold increases for the L. reuteri ΔΦ1 and ΔΦ2 single mutants, respectively (*P > *0.05), while deletion of both prophages increased bacterial counts 3-fold (*P = *0.002). Thus, carriage of prophages reduces the fitness of L. reuteri 6475 during gastrointestinal transit.

### Temporal and spatial scale of phage production of L. reuteri in digestive tract.

In an attempt to determine the impact of each prophage on reducing the fitness of L. reuteri during gastrointestinal transit, we first determined how many phages were produced. Although there was a statistically significant difference, between the L. reuteri COMP and wild-type strains, the two strains still produced very similar amounts of PFU as detected in fecal samples (2.33 × 10^4^ versus 1.11 × 10^4^ PFU/100 mg feces for COMP versus WT, respectively; *P = *0.02) (Fig. 3B). Administration of the L. reuteri ΔΦ2 strain yielded 10-fold more PFU than the L. reuteri wild type (1.48 × 10^5^ versus 1.11 × 10^4^ PFU/100 mg feces for ΔΦ2 mutant versus WT, respectively; *P < *0.001), while no PFU were identified in the feces of animals gavaged with the L. reuteri ΔΦ1 or L. reuteri ΔΦ1 ΔΦ2 mutant.

Understanding where phages are induced during gastrointestinal transit may lead to identification of the trigger(s) of phage production in the intestinal tract. To get a sense of the temporal and spatial scale of phage production in the intestinal tract, we performed a time course experiment whereby the L. reuteri wild type was administered with a single dose of 10^9^ CFU. We analyzed the stomach, three distinct regions in the small intestine (duodenum, jejunum, and ileum), the cecum, the colon, and the feces at 3, 6, and 12 h postgavage. At all three time points, phages were mainly recovered from the ileum and the large intestine, with the highest densities detected in the cecum, colon, and feces (Fig. 3C). These data suggest that the distal intestinal tract (cecum and colon) yields more phage production compared to the small intestinal regions.

### Stomach acid does not induce phage production in the GI tract.

The experiments above established that during GI transit prophages are induced in L. reuteri, which impacts host survival. We aimed next to determine what environmental cues contribute to the findings presented above. During GI transit, bacteria encounter several conditions, including acid stress in the stomach, which could activate prophages. To determine the effect of stomach acids on phage production by L. reuteri, we performed an experiment in which animals were treated with omeprazole, a proton pump inhibitor that increases the pH in the stomach (38), which has been used previously to determine the role of stomach acidity in gut colonization of L. reuteri (39). Animals (*n* = 4/group) were administered with vehicle or with omeprazole for 2 days, followed by administration of 10^9^ bacteria (L. reuteri wild type or L. reuteri ΔΦ1 ΔΦ2 mutant). Supplementation of omeprazole increased gastrointestinal survival of both the L. reuteri wild type (1.73 × 10^7^ versus 4.93 × 10^7^ CFU/100 mg feces for water versus omeprazole, respectively; *P < *0.0001 [Fig. 3D]) and L. reuteri ΔΦ1 ΔΦ2 mutant (5.87 × 10^7^ versus 1.19 × 10^8^ CFU/100 mg feces for water versus omeprazole, respectively; *P = *0.007 [Fig. 3D]). We recovered similar levels of phage following GI transit of the L. reuteri wild type in the omeprazole and water groups (2.05 × 10^3^ versus 3.38 × 10^3^ PFU/100 mg feces for water versus omeprazole, respectively; *P = *0.557 [Fig. 3E]). These data demonstrate that prophages do not influence the survival of L. reuteri in stomach acid and stomach acid is not a major contributor of phage induction.

### *In vivo* phage production is dependent on activation of the SOS response.

Next, we addressed the question of whether L. reuteri phage production during gastrointestinal transit is induced by stress on the bacterial cells. The SOS response is among the best-studied stress-response systems, which induces DNA repair and recombination to promote survival when exposed to stressful conditions. A key player in activation of the SOS response is RecA, which—at least in E. coli—together with the repressor LexA regulates the expression of genes involved in DNA repair (40). Prophages are induced following activation of the SOS response, including L. reuteri (15). However, knowledge is limited to what extent the stress response triggers phage production during gastrointestinal transit. To test this, we used a mutant in which *recA* is inactivated (L. reuteri *ΔrecA*). We administered 10^9^ CFU of the L. reuteri wild type or L. reuteri Δ*recA* to mice, and 2 days after administration we determined the bacterial and viral loads in the feces. Inactivation of *recA* significantly reduced the ability of L. reuteri to survive GI transit (Fig. 3F; 3.42 × 10^6^ versus 8.38 × 10^5^ CFU/100 mg feces for WT versus Δ*recA* mutant, respectively; *P < *0.001), while phage levels following transit of the L. reuteri Δ*recA* mutant were below the detection limit (Fig. 3G). Thus, although our data show that the SOS response likely induces phages during gastrointestinal transit, there are other factors driven by RecA—or the SOS response—that influence fitness independent of phages.

### Production of phages provides L. reuteri with a competitive advantage in the gastrointestinal tract when in direct competition.

**FIG 4:**
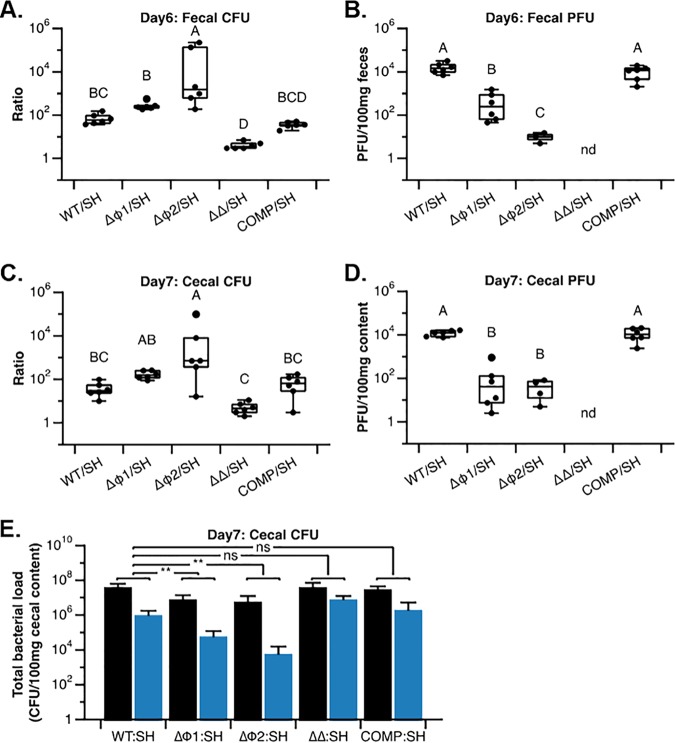
Phages provide L. reuteri with a competitive advantage in the gastrointestinal tract. (A) Competition ratios at day 6 between the L. reuteri wild type and the sensitive host (WT/SH), the ΔΦ1 mutant and the sensitive host (ΔΦ1/SH), the ΔΦ2 mutant and the sensitive host (ΔΦ2/SH), the ΔΦ1 ΔΦ2 mutant and the sensitive host (ΔΔ/SH), and the L. reuteri ΔΦ1 ΔΦ2∷Φ1∷Φ2 complemented strain and the sensitive host (COMP/SH). Bacterial counts of the competing strains (rifampin resistant) and the sensitive host strain (chloramphenicol resistant) were normalized to 100 mg fecal material followed by determination of the ratio. (B) Phage numbers at day 6 following competition between the different competing strains and the sensitive host (see panel A for details). Data are expressed as PFU normalized to 100 mg feces. (C) Competition ratios at day 7 of the competing strains (see panel A for details). Bacterial counts of the competing strains (rifampin resistant) and the sensitive host strain (chloramphenicol resistant) were normalized to 100 mg intestinal content followed by determining the ratio. (D) Phage numbers in the ceca at day 7 following competition between the different competing strains and the sensitive host (see panel A for details). Data are expressed as PFU normalized to 100 mg intestinal content. (A to D) Means with different capital letters indicate statistical significance (ANOVA; *P* < 0.05; Tukey’s HSD test). nd, not detected. (E) Total bacterial counts of the competing strains (black bars) and the sensitive host (SH [blue bars]) at day 7 in the ceca normalized to 100 mg intestinal content. **, *P* < 0.01 as determined by *t* test; ns, not significant.

So far, our findings show that active prophages are widely distributed in the mutualistic host-associated species Lactobacillus reuteri. Using L. reuteri 6475 as our model organism, we found that phages are produced during gastrointestinal transit in a SOS-dependent manner; however, phages reduce the fitness in the main habitat of L. reuteri. This raises the question: why do so many strains—from different lineages—maintain prophages in their genome over evolutionary times despite the fact prophages could be inactivated by mutations or deleted by homologous recombination? We hypothesized that production of phages by L. reuteri provides a competitive advantage by reducing the colonization of a strain that is susceptible to these phages. To test this, we coadministered 1:1 mixtures of the sensitive host with either the L. reuteri wild type, or the three prophage deletion variants (with single or double deletions), or the complemented strain. Each mixture was administered to a group of germfree Swiss-Webster mice (*n* = 6/group). To distinguish between the strains by standard plate counts, we tagged the strains to yield different antibiotic resistance profiles (Table 3). We confirmed that all tagged strains had similar growth rates (Fig. S3B and C). Six days postadministration, fecal material was analyzed for bacterial and phage counts, and animals were sacrificed at day 7, after which we analyzed the cecal contents. Based on the fecal counts, the L. reuteri wild type outcompeted the sensitive host by 74-fold, which was comparable to the competition ratio of the complemented strain (37-fold; Fig. 4A). The L. reuteri ΔΦ1 and ΔΦ2 single mutants outcompeted the sensitive host by 288- and 6.0 × 10^4^-fold, respectively. This fitness advantage was not detectable for the L. reuteri ΔΦ1 ΔΦ2 double mutant, which was recovered in similar proportions to the sensitive host. Thus, in our model system, the prophage-containing strains contribute to the *in vivo* competitive advantage of L. reuteri. While the ratio of the L. reuteri wild-type and complemented strains to the sensitive host was lower than those of the single-prophage-deletion strains, we recovered the highest viral load from the wild-type and the complemented strains (1.64 × 10^4^ versus 1.06 × 10^4^ PFU/100 mg feces for WT versus COMP, respectively [Fig. 4B]). Competition experiments with the L. reuteri ΔΦ1 or ΔΦ2 mutant yielded 502 and 10 PFU/100 mg feces for the ΔΦ1 and ΔΦ2 mutants, respectively. Similar results were obtained when we examined the competition ratios and the viral load in the ceca (Fig. 4C and D).

**TABLE 3:** Bacterial strains used in this study^*a*^

Species	Strain	Description	Reference or source
E. coli	EC1000	*In trans* RepA provider, Kan^r^ (cloning host)	41
	VPL3002	EC1000 harboring pVPL3002, Em^r^	35
	VPL3590	EC1000 harboring pVPL3590, Em^r^	15
	VPL3593	EC1000 harboring pVPL3593, Em^r^	15
	VPL3746	EC1000 harboring pVPL3746, Em^r^	15
	VPL3749	EC1000 harboring pVPL3749, Em^r^	15
	VPL3810	EC1000 harboring pVPL3810, Em^r^	15
	VPL3886	EC1000 harboring pVPL3886, Cm^r^	This study

L. lactis	VPL2042	L. lactis NZ9000 harboring pVPL2042, Em^r^	35

L. reuteri	ATCC PTA 6475	Wild type, human breast milk isolate	BioGaia AB. (Fig. S2)
	VPL4079	VPL1014 ΔLRФ1 Δ*attB1*	15
	VPL4104	VPL1014 ΔLRФ2 Δ*attB2*	15
	VPL4090 (LH)	VPL4079 ΔLRФ2 Δ*attB2*	15
	VPL4119 (ΔФ1)	VPL4079::*attB1*	This study
	VPL4120 (ΔФ2)	VPL4104::*attB2*	This study
	VPL4121 (ΔФ1 ΔФ2)	VPL4150::*attB2*	15
	VPL4126 (WT Rif^r^)	VPL1014 *rpoB*::oVPL236 (T487S H488R) Rif^r^	15
	VPL4132 (ΔФ1 Rif^r^)	VPL4119 *rpoB*::oVPL236 (T487S H488R) Rif^r^	This study
	VPL4135 (ΔФ2 Rif^r^)	VPL4120 *rpoB*::oVPL236 (T487S H488R) Rif^r^	This study
	VPL4129 (ΔФ1 ΔФ2 Rif^r^)	VPL4121 *rpoB*::oVPL236 (T487S H488R) Rif^r^	15
	VPL4150	VPL4121::LRФ1	This study
	VPL4152	VPL4121::LRФ2	This study
	VPL4159 (COMP)	VPL4152::LRФ1	This study
	VPL4154	VPL4129::LRФ1 Rif^r^	This study
	VPL4156	VPL4129::LRФ2 Rif^r^	This study
	VPL4161 (COMP Rif^r^)	VPL4156::LRФ1 Rif^r^	This study
	VPL4178 (LH Cm^r^)	VPL4090::Cm^r^	This study
	VPL4167 (ΔФ1 Cm^r^)	VPL4119::Cm^r^	This study
	VPL4181 (ΔФ2 Em^r^)	VPL4120::Em^r^	This study
	DSM 20016	Human isolate	ATCC (Fig. S2)
	SD2112	Human isolate	BioGaia AB (Fig. S2)
	ATCC PTA 4659	Human isolate	BioGaia AB (Fig. S2)
	PNG008B_M	Human isolate	Jens Walter (Fig. S2)
	PNG008-2c_8_1	Human isolate	Jens Walter (Fig. S2)
	PNG008_48h	Human isolate	Jens Walter (Fig. S2)
	PNG008_24h	Human isolate	Jens Walter (Fig. S2)
	PNG008_ANA	Human isolate	Jens Walter (Fig. S2)
	PNG008A_M	Human isolate	Jens Walter (Fig. S2)
	PNG008C_M	Human isolate	Jens Walter (Fig. S2)
	DSM 20056	Human isolate	JGI 642555135 (Fig. S2)
	mlc3	Mouse isolate	JGI 2506381016 (Fig. S2)
	Lpuph-1	Mouse isolate	JGI 2506381017 (Fig. S2)
	Lr4020	Mouse isolate	31 (Fig. S2)
	100-93	Mouse isolate	31 (Fig. S2)
	6799jm-1	Mouse isolate	31 (Fig. S2)
	6798jm-1	Mouse isolate	31 (Fig. S2)
	ML1	Mouse isolate	31 (Fig. S2)
	one-one	Mouse isolate	31 (Fig. S2)
	L1600-1	Mouse isolate	31 (Fig. S2)
	lpupjm1	Mouse isolate	31 (Fig. S2)
	L1604-1	Mouse isolate	31 (Fig. S2)
	Mouse 2	Mouse isolate	31 (Fig. S2)
	Lr4000	Mouse isolate	BioGaia AB (Fig. S2)
	100-23	Rat isolate	JGI 2500069000 (Fig. S2)
	FUA3043	Rat isolate	31 (Fig. S2)
	FUA3048	Rat isolate	31 (Fig. S2)
	N2D	Rat isolate	Siv Ahrné (Fig. S2)
	N4I	Rat isolate	31 (Fig. S2)
	Rat 19	Rat isolate	31 (Fig. S2)
	R2LC	Rat isolate	Siv Ahrné (Fig. S2)
	CR	Rat isolate	31 (Fig. S2)
	2010	Rat isolate	BioGaia AB (Fig. S2)
	N2J	Rat isolate	Siv Ahrné (Fig. S2)
	AD 23	Rat isolate	31 (Fig. S2)
	bmc2	Rat isolate	Stefan Roos (Fig. S2)
	LK139	Chicken isolate	Jens Walter (Fig. S2)
	11284	Chicken isolate	Jens Walter (Fig. S2)
	KS6	Chicken isolate	Jens Walter (Fig. S2)
	KE1	Chicken isolate	Jens Walter (Fig. S2)
	LB54	Chicken isolate	Jens Walter (Fig. S2)
	1204	Chicken isolate	Jens Walter (Fig. S2)
	LK146	Chicken isolate	Jens Walter (Fig. S2)
	LK20	Chicken isolate	Jens Walter (Fig. S2)
	LK94	Chicken isolate	Jens Walter (Fig. S2)
	LK159	Chicken isolate	Jens Walter (Fig. S2)
	LK75	Chicken isolate	Jens Walter (Fig. S2)
	KYE26	Chicken isolate	Jens Walter (Fig. S2)
	KY21	Chicken isolate	Jens Walter (Fig. S2)
	HWB7	Chicken isolate	Jens Walter (Fig. S2)
	HWH3	Chicken isolate	Jens Walter (Fig. S2)
	HW8	Chicken isolate	Jens Walter (Fig. S2)
	CSA9	Chicken isolate	Jens Walter (Fig. S2)
	CSB7	Chicken isolate	Jens Walter (Fig. S2)
	L1	Chicken isolate	Jens Walter (Fig. S2)
	L2	Chicken isolate	Jens Walter (Fig. S2)
	KL3B	Chicken isolate	Jens Walter (Fig. S2)
	L3S	Chicken isolate	Jens Walter (Fig. S2)
	L4	Chicken isolate	Jens Walter (Fig. S2)
	L5	Chicken isolate	Jens Walter (Fig. S2)
	JCM1081	Chicken isolate	JGI 2684623011 (Fig. S2)
	1366	Chicken isolate	JGI 2684623010 (Fig. S2)
	CSF8	Chicken isolate	JGI 2684623009 (Fig. S2)
	11283	Chicken isolate	Jens Walter (Fig. S2)
	LK150	Chicken isolate	Jens Walter (Fig. S2)
	NCK983	Chicken isolate	Jens Walter (Fig. S2)
	ATCC 53608	Pig isolate	BioGaia AB (Fig. S2)
	I5007	Pig isolate	JGI 2554235423 (Fig. S2)
	LP167-67	Pig isolate	BioGaia AB (Fig. S2)
	3C6	Pig isolate	JGI 2599185333 (Fig. S2)
	I5007	Pig isolate	JGI 2554235423 (Fig. S2)
	3c6	Pig isolate	JGI 2599185333 (Fig. S2)
	53608	Pig isolate	EMBL LN906634 (Fig. S2)
	LP167-67	Pig isolate	JGI 2599185361 (Fig. S2)
	13S14	Pig isolate	Jens Walter (Fig. S2)
	10C2	Pig isolate	Jens Walter (Fig. S2)
	393	Pig isolate	Jens Walter (Fig. S2)
	6S15	Pig isolate	Jens Walter (Fig. S2)
	4S17	Pig isolate	Jens Walter (Fig. S2)
	104R	Pig isolate	Jens Walter (Fig. S2)
	LEM83	Pig isolate	Jens Walter (Fig. S2)
	23012	Pig isolate	Jens Walter (Fig. S2)
	JW2015	Pig isolate	Jens Walter (Fig. S2)
	JW2016	Pig isolate	Jens Walter (Fig. S2)
	JW2017	Pig isolate	Jens Walter (Fig. S2)
	JW2019	Pig isolate	Jens Walter (Fig. S2)
	20/2	Pig isolate	JGI 2599185332 (Fig. S2)
	27/4	Pig isolate	Jens Walter (Fig. S2)
	69/3	Pig isolate	Jens Walter (Fig. S2)
	146/2	Pig isolate	Jens Walter (Fig. S2)
	173/3	Pig isolate	Jens Walter (Fig. S2)
	173/4	Pig isolate	Jens Walter (Fig. S2)
	173/5	Pig isolate	Jens Walter (Fig. S2)
	32	Pig isolate	Jens Walter (Fig. S2)
	676	Pig isolate	Jens Walter (Fig. S2)
	1704	Pig isolate	Jens Walter (Fig. S2)
	1013	Pig isolate	Jens Walter (Fig. S2)
	1048	Pig isolate	Jens Walter (Fig. S2)
	1068	Pig isolate	Jens Walter (Fig. S2)
	1063	Pig isolate	Jens Walter (Fig. S2)
	LPA1	Pig isolate	Jens Walter (Fig. S2)
	Cp415	Pig isolate	Jens Walter (Fig. S2)
	Cp447	Pig isolate	Jens Walter (Fig. S2)
	P26	Pig isolate	Jens Walter (Fig. S2)
	P97	Pig isolate	Jens Walter (Fig. S2)

a Rif^r^, rifampin resistant; RpoB, DNA-directed RNA polymerase (HMPREF0536_0828 for L. reuteri); Cm^r^, chloramphenicol resistant; Em^r^, erythromycin resistant; VPLxxxx, van Pijkeren Lab culture collection identification number. JGI and EMBL numbers are found at the Joint Genome Institute (JGI) genome portal (http://genome.jgi.doe.gov) and the European Molecular Biology Laboratory (https://www.embl.de/), respectively.

In an effort to determine why the L. reuteri wild type versus sensitive host and L. reuteri ΔΦ2 mutant versus sensitive host yielded the lowest and highest competition ratio yet yielded the most and fewest phage, respectively, we compared the total cecal bacterial load in each of these competition experiments. The total bacterial load of competitions between the wild-type and the sensitive host was 8-fold higher than competitions between the L. reuteri ΔΦ2 mutant and the sensitive host (Fig. 4E), which leads us to suggest that differences in the size of the bacterial community could explain differences in the phage population. Collectively, our data demonstrate that phage production by the gut symbiont Lactobacillus reuteri provides the organism with a competitive advantage in the gut when in competition with a strain that competes for the same nutrients.

## DISCUSSION

In this work, we demonstrated that active prophages are present in the majority of Lactobacillus reuteri strains originating from different vertebrate hosts. Phages are produced throughout the gastrointestinal tract, which appears to be dependent on activation of the SOS response. In addition, we established that prophages have a double-edged sword effect on the fitness of this mutualistic microbe in the gut ecosystem.

Nearly all cellular life forms harbor parasites, which includes bacteria harboring prophages (42). Metagenome analysis of human feces revealed that the bacteriophage community is remarkably stable, which—combined with a low virus-to-microbe ratio in the gut—collectively reflects a lysogenic lifestyle (3, 43, 44). A study by Kim and Bae investigating the mouse microbiota also revealed that lysogeny is prevalent and widely distributed, where a large fraction of commensal lysogens were identified as *Firmicutes* (45). Indeed, nearly all human- and rodent-derived L. reuteri isolates we tested (*n* = 37) contained active prophages, but a lower distribution of active prophages was observed in chicken (63%) and pig (44%) lineages. In hog and poultry farming, antibiotics are broadly used to treat and prevent disease and to improve the animal’s growth rate (46). Several antibiotics, including carbadox and ASP250, are known to induce prophages in the gastrointestinal tract of swine (47, 48). Thus, extensive antibiotic use may have selected for L. reuteri strains that did not harbor, have lost, or contain a cryptic prophage (a defective prophage that cannot enter the lytic cycle). For example, the pig-derived L. reuteri strains I5007 and LP167-67 do contain a presumptive intact prophage (Table 1), yet mitomycin C treatment did not lead to cell lysis, which indeed suggests these prophages are cryptic. While we cannot exclude that prophages are induced via a mechanism other than the SOS pathway, overall, we provided experimental evidence that active prophages are broadly distributed in the gut symbiont species Lactobacillus reuteri (69% prevalence). This suggests that prophages are important for this mutualistic microbe because they are evolutionary conserved.

Despite their assumed importance, we established that in conventional mice, prophage carriage comes at a fitness cost. Similar observations were made in a study by De Paepe et al., who found that phage λ of E. coli was induced in the intestine of monoxenic mice, which consequently reduced the fitness of the lysogen (14). Their finding was elegantly validated by a strain with a mutation in the phage repressor—abolishing phage induction—that was fitter than the inducible strain (14). Another example where prophage carriage can be a burden has been established in Staphylococcus aureus. In the nasopharynx—the upper part of the throat behind the nose—S. aureus is replaced the hydrogen-peroxide producing microbe Streptococcus pneumoniae (49). Exposure of S. aureus to H_2_O_2_ activates its prophages, which consequently reduces the fitness of S. aureus and which allows the H_2_O_2_-resistant S. pneumoniae to invade the niche. Thus, both host-derived as well as microbe-derived triggers can activate prophages in a lysogen thereby reducing fitness.

In an attempt to understand the mechanism by which L. reuteri prophages are induced in the intestine, we found that prophage production in the gut is strictly dependent on activation of the SOS response. While exposure to a low pH can activate prophages (50, 51), this does not seem to be a conserved mechanism. We found that the acidity in the stomach does not contribute to L. reuteri phage production, which is in-line with previous observations in Lactococcus lactis where *in vitro* growth in media with a lower pH did not boost prophage induction (52). Thus, while the stomach environment reduced L. reuteri survival, this does not seem to trigger activation of the SOS response leading to phage production. This means that other mechanisms are at play, potentially driven by exposure to short-chain fatty acids (SCFAs). Throughout the intestine, the dominant SCFAs are acetic acid, butyric acid, and propionic acid (53). We previously demonstrated that exposure to biologically relevant levels of SCFAs activates the Ack pathway, which accordingly activates the SOS response, leading to a 3-log increase in phage production (15). These, and potentially other, stressors could drive phage production in L. reuteri, thereby reducing the fitness in the gut ecosystem.

Now we have established that the gut environment activates the SOS response leading to prophage production and reduced fitness, why are lysogens dominant in the gut ecosystem (3, 8, 43–45) while evidence exists that prophages can be lost (54, 55)? Carriage of prophages can alter the biology of their host and potentially provides the host with an advantage (reviewed in references 56 and 57). Briefly, prophages can alter cellular transcription (58), introduce new functions such as immunity to infection by other phages (59), promote DNA transfer that allows the cell to acquire, for example, antibiotic resistance (60), and induced prophages can provide the host with a competitive advantage by killing competitor strains (27). In our study, we tested the hypothesis that phage production provides the gut symbiont Lactobacillus reuteri with a competitive advantage by killing a competitor strain. We found that L. reuteri wild type—harboring two prophages—outcompeted the sensitive host less efficiently than the single-prophage-deletion strains did. A study in E. coli investigated within-host competition of prophages and identified that the presence of multiple prophages can reduce lytic productivity of the lysogen (61), which could explain our observed differences in competition ratios. Interestingly, in each of our competition experiments, we observed that the sensitive strain is outcompeted, yet, not eradicated by the phage-producing strain. This could indicate there is frequency-dependent selection (62), meaning that the sensitive strain seems to determine the amount of phage present. If the cell density of the sensitive host is reduced, then phage levels are reduced. Perhaps, this explains in our competition experiments why most phages are recovered from communities that contain the densest population of the sensitive host and why the sensitive host does not go extinct.

In this work, we used carefully controlled experiments with genetically modified strains to elucidate the nature of the interrelationship of prophages with a bacterial gut symbiont. Although prophages are induced and produced in the gut and thereby display a fitness burden to their host, the relationship between L. reuteri and the prophage cannot primarily be described as parasitism. Instead, prophages can provide L. reuteri with a competitive advantage against direct competitors (e.g., strains of the same species), suggesting an element to prophages that supports a mutualistic relationship with its host. Overall, these findings provide potential explanations for why bacterial gut symbionts maintain prophages in their genomes and the large amount of prophage-derived virus particles in gut microbiomes. The findings are relevant as they provide basic information on how host-phage interplay influences bacterial interactions in gut ecosystems. Future work should determine if fitness trade-offs associated with bearing prophages can stabilize the coexistence of sensitive and resistant strains and thus contribute to strain diversity in gut ecosystems. Lastly, increased knowledge of prophage-mediated lysis in the intestinal tract will be important to continue the development of recombinant L. reuteri to deliver therapeutics in the gut following prophage-mediated lysis (63, 64).

## MATERIALS AND METHODS

### Bacterial strains and growth conditions.

The strains and plasmids used in this study are listed in Table 3 and Table 4. All L. reuteri strains were cultured at 37°C in deMan-Rogosa-Sharpe medium (MRS; BD BioSciences). Escherichia coli and Lactococcus lactis strains were cultured in lysogeny broth (LB [Teknova]) and M17 (BD BioSciences), respectively. M17 broth was supplemented with 0.5% (wt/vol) glucose. If applicable, antibiotics were added as follows: erythromycin and chloramphenicol were supplemented at 5 μg/ml for L. reuteri and L. lactis strains, and rifampin or vancomycin was supplemented at 25 μg/ml or 500 μg/ml, respectively, for L. reuteri. E. coli was supplemented with 300 μg/ml or 20 μg/ml erythromycin or chloramphenicol, respectively.

**TABLE 4:** Plasmids and bacteriophages used in this study^*a*^

Plasmid or bacteriophage	Genotype	Description	Source
Plasmids			
pVPL2042	pNZ8048::Em^r^		This study
pVPL3002	pORI19::*ddlA* F258Y_reuteri_, Em^r^	Suicide shuttle vector with vancomycin counterselection marker	35
pVPL3048	pVPL3002::gene insertion cassette (PCR with oVPL265-266), Em^r^		This study
pVPL3590	pVPL3002::LRФ1 deletion cassette, Em^r^	Deletion cassette targets entire LRФ1 and *attB1*	15
pVPL3593	pVPL3002::LRФ2 deletion cassette, Em^r^	Deletion cassette targets entire LRФ2 and *attB2*	15
pVPL3746	pVPL3002::*attB1* recovery cassette, Em^r^	For *attB1* recovery on ΔLRФ1 and Δ*attB1* background	15
pVPL3749	pVPL3002::*attB2* recovery cassette, Em^r^	For *attB2* recovery on ΔLRФ2 and Δ*attB2* background	15
pVPL3810	pVPL3002::Cm^r^ gene insertion cassette, Em^r^	For Cm^r^ gene insertion in L. reuteri VPL1014 genome	This study
pVPL3886	pVPL3002::Em^r^ gene insertion cassette, Cm^r^	For Em^r^ gene insertion in L. reuteri VPL1014 genome	This study
pJP028	pNZ8048::P_help_::Cm^r^ Em^r^		Lab stock
pNZ8048	Nisin-inducible promoter, Cm^r^		65
Bacteriophages			
VPL1014 Ф1 (LRФ1)	LAR0766∼LAR0809 in L. reuteri JCM1112 reference genome	LRФ1 was isolated from VPL4120 (GenBank accession no. MH837542 [https://www.ncbi.nlm.nih.gov/genome/?term=MH837542])	15
VPL1014 Ф2 (LRФ2)	LAR1081∼LAR1041 in L. reuteri JCM1112 reference genome	LRФ2 was isolated from VPL4119 (GenBank accession no. MH837543 [https://www.ncbi.nlm.nih.gov/genome/?term=MH837543])	15

a Cm^r^, chloramphenicol resistant; Em^r^, erythromycin resistant; pVPLxxxx, van Pijkeren Lab plasmid collection identification number; DdlA, d-alanine-d-alanine ligase (HMPREF0536_1572).

### Reagents and enzymes.

DNA fragments were cloned by ligation cycle reaction (LCR) (66). Prior to electroporation, LCRs were precipitated with Pellet Paint (Novagen). For cloning purposes or screen purposes, we used Phusion Hot Start polymerase II (Fermentas) or *Taq* polymerase (Denville Scientific), respectively. For standard ligations, we used T4 DNA ligase (Thermo Scientific). Oligonucleotides and synthetic double-stranded DNA fragments were synthesized by Integrated DNA Technologies (IDT), and are listed in Table 5.

**TABLE 5:** Oligonucleotides used in this study

Oligonucleotide	Sequence (5′→3′)^*a*^	Description^*b*^
oVPL49	ACAATTTCACACAGGAAACAGC	Oligo paired with oVPL97 used for screening pVPL3002 constructs
oVPL97	CCCCCATTAAGTGCCGAGTGC	Oligo paired with oVPL49 used for screening pVPL3002 constructs
oVPL187	TACCGAGCTCGAATTCACTGG	Rev, internal oligo for pVPL3002 backbone amplification
oVPL188	ATCCTCTAGAGTCGACCTGC	Fwd, internal oligo for pVPL3002 backbone amplification
oVPL202	ATGAACTTTAATAAAATTGATTTAGAC	Fwd, Cm^r^ gene from pNZ8048
oVPL203	TTATAAAAGCCAGTCATTAGGCC	Rev, Cm^r^ gene from pNZ8048
oVPL236	TCAAACCACCAGGACCAAGCGCTGAAAGACGACGCTTtctgcTTAATTCACCTAATGGGTTGGTTTGATCCATGAACTGG	Recombineering oligos targeting *L. reuteri* VPL1014 RpoB gene
oVPL265	TCTGTGGGGATACACTGCGATTACATG	Fwd, paired with oVPL266 used for Em^r^ and Cm^r^ gene insertion cassette (u/s and d/s)
oVPL266	ACTATGCTGATGGAATTGATACTAGCTGG	Rev, paired with oVPL265 used for Em^r^ and Cm^r^ gene insertion cassette (u/s and d/s)
oVPL271	TTAAAAATTAATCTTTCCAGTAATAATCAACATC	Fwd, internal oligo for pVPL3048 backbone for cloning Em^r^ and Cm^r^ genes
oVPL272	TTAAAATGTAGGTTTAATTTTTAGGGC	Rev, internal oligo for pVPL3048 backbone for cloning Em^r^ and Cm^r^ genes
oVPL279	TGCGCTGATGTTGATTATTACTGGAAAGATTAATTTTTAACATTATGCTTTGGCAGTTTATTCTTGACATG	Fwd, paired with oVPL280 for P_help_::Cm^r^ gene amplification from pJP028
oVPL280	AAGCAGTCAAAAAGCCCTAAAAATTAAACCTACATTTTAATTTGATTGATAGCCAAAAAGCAGCAG	Rev, paired with oVPL279 for P_help_::Cm^r^ gene amplification from pJP028
oVPL304	AACAGCTTGCCGTTGCATGTTAGC	Oligo paired with oVPL305, oVPL306 for MAMA PCR screening of *L. reuteri* VPL1014 *rpoB* recombinants
oVPL305	AAAAGGGTGATACGGTAACCAAGG	Oligo paired with oVPL304, oVPL306 for MAMA PCR screening of *L. reuteri* VPL1014 *rpoB* recombinants
oVPL306	AAGCGCTGAAAGACGACGCTTTCTG	Oligo paired with oVPL304, oVPL305 for MAMA PCR screening of *L. reuteri* VPL1014 *rpoB* recombinants
oVPL334	AACTTTCGCCATTAATGTGTTTTATCGG	Fwd, for SCO and DCO screening of Cm^r^ and Em^r^ gene insertion
oVPL335	AGACAGATGACAAGCCCTTTAGC	Rev, for SCO and DCO screening of Cm^r^ and Em^r^ gene insertion
oVPL1377	TGCCCGTAATTTGCGAGTTC	Fwd, internal of *recT1* in LRФ1
oVPL1378	ACAGGTTGCCAATCCTCTTTG	Rev, internal of *recT1* in LRФ1
oVPL1379	GCTAGAAACGGGTTGCCAAT	Fwd, internal of *recT2* in LRФ2
oVPL1380	TCTACCTGCTGATGTTATGGGA	Rev, internal of *recT2* in LRФ2
oVPL1390	TAAGTTAAGGGATGCATAAACTGCATCC	Fwd, internal oligo for pVPL3048 backbone for cloning Cm^r^ gene
oVPL1391	TATAACCCTCTTTAATTTGGTTATATG	Rev, internal oligo for pVPL3048 backbone for cloning Cm^r^ gene
oVPL1436	AGGATTCCGACAACGTGACT	Fwd, screening oligo paired with oVPL1438 used for screening LRФ1 excision after MitC induction, Fwd, for SCO and DCO screening of LRФ1deletion
oVPL1437	AGTAGCGACGGCGATTAAGA	Fwd, screening oligo paired with oVPL1439 used for screening LRФ1 excision after MitC induction
oVPL1438	TATGCTGCGCTCAGTAATGG	Rev, screening oligo paired with oVPL1436 used for screening LRФ1 excision after MitC induction
oVPL1439	ATCTGCCATTGTTGCTTTCC	Rev, screening oligo paired with oVPL1437 used for screening LRФ1 excision after MitC induction, Rev, oligo SCO and DCO screening of LRФ1 deletion
oVPL1440	AGATGTTATTTCAGCGGTGGCG	Fwd, screening oligo paired with oVPL1441 used for screening LRФ2 excision after MitC induction
oVPL1441	AATGCCACGAGGATTGATCGGG	Rev, screening oligo paired with oVPL1440 used for screening LRФ2 excision after MitC induction
oVPL1442	ACTTAAAAACTGAGCAGCAATTGC	Sequencing oligo starting 49 bases d/s of oVPL1440
oVPL1443	ATTATAACTCCAATATAATTTTCGCGC	Sequencing oligo starting 398 bases d/s of oVPL1440
oVPL1444	TTGGAACAAAGTGAAGGTCTTTAATTGC	Sequencing oligo starting 53 bases d/s of oVPL1436
oVPL1445	ATAATTGAGTTACTACCAAATTGGTGAGC	Sequencing oligo starting 522 bases d/s of oVPL1436
oVPL1446	AAACGGAGATACCGAATTTAGGC	Sequencing oligo starting 41 bases d/s of oVPL1437
oVPL1516	AATTGGTGAGCCATTGGAAC	Fwd, 978 bp 5' upstream flanking sequence of LRФ1 omitting *attB1L* sequence
oVPL1517	TGGGATCGGGGGCCAGCTTGGAC	Rev, 978 bp 5' upstream flanking sequence of LRФ1 omitting *attB1L* sequence
oVPL1518	TGTAAGTGGTAAGCCGAGTAAC	Fwd, 1,257 bp 5' downstream flanking sequence of LRФ1 omitting *attB1R* sequence
oVPL1519	TGGCTCAACAAGACACAAGC	Rev, 1,257 bp 5' downstream flanking sequence of LRФ1 omitting *attB1R* sequence
oVPL1520	AAACGACGGCCAGTGAATTCGAGCTCGGTAAATTGGTGAGCCATTGGAACAACAAGCCCG	Bridging oligo for LCR assembly to construct pVPL3590
oVPL1521	GCTAAGTGTCCAAGCTGGCCCCCGATCCCATGTAAGTGGTAAGCCGAGTAACAAACCAAT	Bridging oligo for LCR assembly to construct pVPL3590
oVPL1522	AACCTTATCTGCTTGTGTCTTGTTGAGCCAATCCTCTAGAGTCGACCTGCAGGCATGCAA	Bridging oligo for LCR assembly to construct pVPL3590
oVPL1528	ATCGCAGCCTTAAGGAAATG	Fwd, 1,217 bp 5' upstream flanking sequence of LRФ2 omitting *attB2L* sequence
oVPL1529	AGAAGTACCGGCATGCAAAG	Rev, 1,217 bp 5' upstream flanking sequence of LRФ2 omitting *attB2L* sequence
oVPL1530	ACCAGTACGTTCACGTAAGTAG	Fwd, 1,139 bp 5' downstream flanking sequence of LRФ2 omitting *attB2R* sequence
oVPL1531	GATGCGATTGCGGCTAATAC	Rev, 1,139 bp 5' downstream flanking sequence of LRФ2 omitting *attB2R* sequence
oVPL1532	AAACGACGGCCAGTGAATTCGAGCTCGGTAATCGCAGCCTTAAGGAAATGGGAGACTTTT	Bridging oligo for LCR assembly to construct pVPL3593
oVPL1533	ACTGAATCCACTTTGCATGCCGGTACTTCTACCAGTACGTTCACGTAAGTAGTAGAGCTT	Bridging oligo for LCR assembly to construct pVPL3593
oVPL1534	CGTTTCAGCGGTATTAGCCGCAATCGCATCATCCTCTAGAGTCGACCTGCAGGCATGCAA	Bridging oligo for LCR assembly to construct pVPL3593
oVPL1585	CGAATCGACCCTGCTAAGTT	Fwd, oligo SCO and DCO screening of LRФ2 deletion
oVPL1586	GCCTTAACTGGTGGGTTTGA	Rev, oligo SCO and DCO screening of LRФ2 deletion
oVPL1716	TGCAAAGGTTCTTGATGCTG	Fwd, Em^r^ gene from pNZ8048_Em^r^
oVPL1717	CCGTTTATTATGCTCGCGTTA	Rev, Em^r^ gene from pNZ8048_Em^r^
oVPL2414	TTGATTATTACTGGAAAGATTAATTTTTAATGCAAAGGTTCTTGATGCTGAAACGGGGGA	Bridging oligo for LCR assembly to construct pVPL3886
oVPL2415	TATTGTCGATAACGCGAGCATAATAAACGGTTAAAATGTAGGTTTAATTTTTAGGGCTTT	Bridging oligo for LCR assembly to construct pVPL3886
oVPL2529	ATTCATATAACCAAATTAAAGAGGGTTATAATGAACTTTAATAAAATTGATTTAGACAAT	Bridging oligo for LCR assembly to construct pVPL3886
oVPL2530	TCAGATAGGCCTAATGACTGGCTTTTATAATAAGTTAAGGGATGCATAAACTGCATCCCT	Bridging oligo for LCR assembly to construct pVPL3886
oVPL3148	CAACGCCGACCAAACTTATT	Fwd, screening oligo paired with oVPL1439 used for screening *L. reuteri* 6475 and DSM 20016T LRФ1 excision
oVPL3150	TGCTTCTGATATTGCCAACG	Fwd, screening oligo paired with oVPL1586 used for screening *L. reuteri* 6475 and DSM 20016T LRФ2 excision
oVPL3597	GCTGCAGCTCACTTTGGATT	Fwd, screening oligo paired with oVPL3599 used for screening *L. reuteri* R2lc Ф1 excision
oVPL3599	TGCTGACCATTGGGATAGTG	Rev, screening oligo paired with oVPL3597 used for screening *L. reuteri* R2lc Ф1 excision
oVPL3600	TCATCGGTGCAGTACTTTGC	Fwd, screening oligo paired with oVPL3601 used for screening *L. reuteri* JCM 1081 Ф1 excision
oVPL3601	ATTCCGTGCCGGTGATACTA	Rev, screening oligo paired with oVPL3600 used for screening *L. reuteri* JCM 1081 Ф1 excision
oVPL3605	GTAAGCGACTAGGCCATCCA	Fwd, screening oligo paired with oVPL3601 used for screening *L. reuteri* JCM 1081 Ф2 excision
oVPL3607	AACACGCCGAAAGTAGTTGG	Rev, screening oligo paired with oVPL3600 used for screening *L. reuteri* JCM 1081 Ф2 excision
oVPL3608	GGTGAACCAACGCTCTCAAT	Fwd, screening oligo paired with oVPL3601 used for screening *L. reuteri* JCM 1081 Ф3 excision
oVPL3609	GTTGGATCATCTCAGCGTCA	Rev, screening oligo paired with oVPL3600 used for screening *L. reuteri* JCM 1081 Ф3 excision
oVPL3626	GGTACGGAGGATGTCGTTGT	Fwd, screening oligo paired with oVPL3601 used for screening *L. reuteri* 2010 Ф1 excision
oVPL3627	CTGTTCCGCATATCCAATCA	Rev, screening oligo paired with oVPL3600 used for screening *L. reuteri* 2010 Ф1 excision
oVPL3628	CAGGGCTTGGATATTGGAGA	Fwd, screening oligo paired with oVPL3601 used for screening *L. reuteri* Lr4020 Ф1 excision
oVPL3629	TTAGCAAGCGGCTAGGTCAT	Rev, screening oligo paired with oVPL3600 used for screening *L. reuteri* Lr4020 Ф1 excision

a Sequence letters in lowercase are the sequence changes for the recombineering oligonucleotide that are identical to the lagging strand of DNA.

b pVPLxxxx, van Pijkeren Lab oligonucleotide collection identification number; oligo, oligonucleotide; d/s, downstream; u/s, upstream; SCO, single crossover; DCO, double crossover; Fwd, forward; Rev, reverse; MAMA-PCR, mismatch amplification mutation assay PCR.

### Bioinformatic analyses.

Prophages in L. reuteri genomes were identified using the PHAge Search Tool (PHASTER) (67). To accomplish this, L. reuteri genome sequences, retrieved from the databases Integrated Microbial Genomes (IMG) at Joint Genome Institute (JGI) and National Center for Biotechnology Information (NCBI), were uploaded to PHASTER followed by standard analysis. Prophages identified in L. reuteri ATCC PTA 6475 were manually annotated following a nonredundant search against the DNA database at NCBI. To compare both prophage genomes in L. reuteri ATCC PTA 6475, we used the Mauve alignment tool (at the default progressive alignment setting) (68).

### Prophage induction.

Overnight cultures of L. reuteri were transferred to an optical density at 600 nm (OD_600_) of 0.1 in 40 ml prewarmed MRS. At an OD_600_ of 0.2 to 0.3, mitomycin C (0.5 μg/ml) was added, and we determined the OD_600_ every hour for up to 8 h. Subsequently, we harvested the bacterial supernatants containing presumptive phages by centrifugation (1 min at 5,000 rpm), followed by filtration (0.22-μm-pore polyvinylidene difluoride [PVDF] filter [Millipore]).

### Identification of prophage *attB* sites in L. reuteri 6475.

Prophage excision in L. reuteri 6475 yielded PCR amplicons with oligonucleotide pairs oVPL1436-1438 and oVPL1437-1439, which flank prophage 1 (LRΦ1), and oVPL1440-1441, which flank prophage 2 (LRΦ2). Following Sanger sequencing (GeneWiz), we determined the *attB* sites based on the presence of palindrome repeats.

### Determination of prophage excision following mitomycin C induction.

Presumptive prophage genomes in select L. reuteri genome sequences were identified with PHASTER. Oligonucleotides were designed that flanked the predicted *attB* sites. L. reuteri strains were subjected to mitomycin C (0.5 μg/ml) at an OD_600_ of 0.3. Two hours following induction, cells were subjected to PCR using oligonucleotide pairs oVPL3148-1439 and oVPL3150-1586 for L. reuteri 6475 or DSM 20016T, oVPL3597-3599 for L. reuteri R2lc, oVPL3600-3601, oVPL3605-3607, and oVPL3608-3609 for L. reuteri JCM 1081, oVPL3626-3627 for L. reuteri 2010, and oVPL3628-3629 for L. reuteri Lr4020 (Table 5).

### Construction of LRΔΦ1, LRΔΦ2, and LRΔΦ1Φ2.

To generate markerless deletions in L. reuteri, we used the recently in-house-developed counterselection plasmid pVPL3002, which is broadly applicable in lactic acid bacteria (35). pVPL3002 is a derivative of pORI19 (69) and encodes the dipeptide ligase enzyme d-Ala-d-Ala (Ddl), which modifies the peptidoglycan cell wall, which consequently increases the binding affinity of vancomycin in bacteria that are intrinsically resistant to vancomycin. Thus, the presence of Ddl (e.g., following single-crossover recombination) reduces the MIC to vancomycin of bacteria that are intrinsically resistant to vancomycin. Cells that have undergone a second homologous recombination event lose the plasmid harboring the *ddl* gene, regain their vancomycin resistance, and have either the wild-type or recombinant genotype. First, we delete each prophage, including the *attB* sites. Briefly, by LCR we cloned the upstream and downstream flank of each prophage in pVPL3002 to yield pVPL3590 and pVPL3593 to delete LRΦ1 and LRΦ2, respectively (see Table 5 for oligonucleotides). Five micrograms of each plasmid was electroporated in L. reuteri 6475 as described previously (35), followed by recovery in MRS and plating on MRS agar containing 5 μg/ml erythromycin. Erythromycin-resistant (Em^r^) colonies were screened by colony PCR to confirm single-crossover homologous recombination using oligonucleotides oVPL49-1436-1439/97-1436-1439 and oVPL49-1585-1586/97-1585-1586 (upstream/downstream) for pVPL3590 and pVPL3593, respectively. Upon confirmation of single-crossover homologous recombination, a single colony was cultured in MRS for 20 generations and plated on MRS agar containing 500 μg/ml vancomycin, which yields colonies only after a second homologous recombination event. Deletion of LRΔΦ1 Δ*attB* and LRΔΦ2 Δ*attB* was confirmed with oligonucleotides oVPL1436-oVPL1439 and oVPL1585-1586, respectively, and the strains were named VPL4079 and VPL4101, respectively. To generate a double-prophage-deletion variant (LRΔΦ1 Δ*attB* ΔΦ2 Δ*attB*), we used LRΔΦ1 Δ*attB* as our genetic background and deleted LRΦ2 and its *attB* site in a manner identical to that described above to yield strain VPL4090. Each deletion variant was subjected to colony purification (3 times), and by PCR, we verified that each strain was cured from the corresponding phage. Next, we restored the *attB* sites of each phage to yield a genotype that is identical to when the phage would naturally excise from the genome. Cell pellets derived from mitomycin C-induced wild-type cells were used as a template for PCR to amplify the *attB*Φ*1* recovery cassette (oVPL1516-1519) and *attB*Φ*2* recovery cassettes (oVPL1528-1531). Each recovery cassette was cloned into the pVPL3002 by LCR with bridging oligonucleotides oVPL1520 and oVPL1522 (*attB*Φ*1*) and oVPL1532 and oVPL1534 (*attB*Φ*2*) to yield pVPL3746 and pVPL3749. In a manner identical to that described above, we restored by two-step homologous recombination the *attB* sites in VPL4079, VPL4101, and VPL4090. To identify single-crossover and double-crossover recombination, we used the same oligonucleotides as for the construction of VPL4079 and VPL4101. The resultant double-crossover recombinants were named VPL4119 (LRΔΦ1), VPL4120 (LRΔΦ2), and VPL4121 (LRΔΦ1 ΔΦ2). A schematic of all strain constructions is displayed in Fig. S1 in the supplemental material.

### Plaque assay for L. reuteri 6475 phages.

Unlike the LRΔΦ1 ΔΦ2 strain, the LRΔΦ1 Δ*attB* ΔΦ2 Δ*attB* strain forms plaques when exposed to LRΦ1 or LRΦ2. Therefore, we used this strain as a sensitive host to determine the PFU/ml using an MRS double-layer agar method. For plaque assays, we followed a method established in a previous study (15).

### Restoring the prophages in LRΔΦ1 ΔΦ2.

Mitomycin C-induced supernatants derived from LRΔΦ2 or LRΔΦ1 contain phages LRФ1 or LRФ2, respectively. LRΔФ1 ΔФ2 (VPL4121) was harvested at an OD_600_ of 4.0, washed once with phage diluent, and resuspended to approximately 2 × 10^9^ CFU/ml. LRΔФ1 ΔФ2 cells were mixed with LRΦ1 supernatant at a 1:1,000 CFU/PFU ratio and incubated for 1 h at 37°C. After infection, cells were harvested by centrifugation (5,000 rpm, 1 min) and resuspended in 1 volume of MRS followed by 3 h of incubation at 37°C. Cells were serially diluted and plated on an MRS agar plate. To assess for the presence of prophage LRΦ1 in LRΔФ1 ΔФ2 following infection, we performed colony PCR (oVPL1377-1378) to amplify part of the LRΦ1 *recT* gene. Next, double-purified colonies that tested positive for the presence of LRΦ1 were subjected to colony PCR (oVPL1436-1439 and oVPL1377-1378) to confirm integration of LRΦ1 in the *attB* site. Mitomycin C induction was performed to assess prophage induction. The strain in which we recovered LRΦ1 in LRΔФ1 ΔФ2 was named VPL4150. Next, we infected VPL4150 with LRΦ2 in a manner identical to that described above. We used oligonucleotide pair oVPL1379-1380 to target the internal region of LRΦ2 *recT2*. Insertion of LRΦ2 in the corresponding *attB* site was confirmed by PCR using oligonucleotides oVPL1440-1441 and oVPL1379-1380. The derivative of LRΔФ1 ΔФ2 in which we restored both LRΦ1 and LRΦ2 was named VPL4159, and is also referred to as the complemented (COMP) strain (Fig. S1).

### Whole-genome sequencing of L. reuteri strains.

Bacterial genome sequencing was performed by the University of Wisconsin—Madison Biotechnology Center. Genomic DNAs were prepared with the Wizard genomic DNA purification kit (Promega) and quantified by Qubit (Life Technologies). DNA libraries were run on the Illumina MiSeq system with 2 × 250-bp reads at the University of Wisconsin—Madison Biotechnology Center. Samples were prepared per the TruSeq Nano DNA LT Library Prep kit (Illumina Inc.) with minor modifications. Samples were sheared using a Covaris M220 ultrasonicator (Covaris, Inc.) and were size selected for an average insert size of 550 bp using SPRI bead-based size exclusion. The quality and quantity of the finished libraries were analyzed using an Agilent DNA1000 chip and Qubit double-stranded DNA (dsDNA) HS assay kit. Libraries were standardized to 2 nM. Paired end, 250-bp sequencing was performed using the Illumina MiSeq Sequencer and a MiSeq 500 bp (v2) sequencing cartridge. Images were analyzed using the standard Illumina Pipeline, version 1.8.2. To identify single nucleotide polymorphisms (SNPs) and indels, comparative genome analysis was performed in SegMan Pro (DNASTAR). All SNPs identified were subjected to Sanger sequencing analysis, and the oligonucleotides for this analysis are listed in Table 5.

### Construction of rifampin-resistant derivatives.

To render rifampin-resistant derivatives of the L. reuteri wild-type strain, the LRΔΦ1, LRΔΦ2, and LRΔΦ1 ΔΦ2 mutant strains, and the complemented strain, we performed single-stranded DNA recombineering (SSDR). Bacteria were transformed with 100 μg oVPL236 to modify *rpoB*. SSDR was performed as described before (34), with the exception that we did not make use of heterologous expression of RecT. Recombinant cells were screened on MRS containing 25 μg/ml rifampin. The genotype of rifampin-resistant colonies was confirmed by mismatch mutation assay PCR (MAMA-PCR) (oVPL304-305-306).

### Cm^r^ gene insertion in L. reuteri sensitive host and ΔФ1.

To insert the gene encoding chloramphenicol resistance (Cm^r^), we first amplified the flanking sequence of a noncoding region from the L. reuteri wild type with oVPL265-266. By standard blunt-end ligation, the amplicon was fused to the pVPL3002 backbone, which was generated with oVPL187-188 to yield pVPL3048. Subsequently, the pVPL3048 backbone was amplified with oVPL271-272 and the P_help_::Cm^r^ cassette amplified from pJP028 (unpublished) with oVPL279-280 followed by Gibson assembly (70) to yield pVPL3810. After transforming the L. reuteri sensitive host and LRΔФ1 with 5 μg pVPL3810, we confirmed single-crossover recombinants with oligonucleotide combinations oVPL203-334-335 (upstream single crossover) and oVPL202-334-335 (downstream single crossover). Double-crossover recombinants were confirmed by PCR (oVPL334-335) and confirmation of erythromycin-sensitive and chloramphenicol-resistant phenotypes. The Cm^r^ mutants of the sensitive host and LRΔФ1 were named VPL4178 (sensitive host Cm^r^) and VPL4167 (LRΔФ1 Cm^r^), respectively (Fig. S1).

### Em^r^ gene insertion in LRΔФ2.

To insert the gene encoding erythromycin resistance (Em^r^), we amplified two fragments of pVPL3048 plasmid backbone with oVPL271-1391 and oVPL272-1390 to omit the Em^r^ gene and Cm^r^ gene expression cassette. The Cm^r^ gene was amplified from pNZ8048 with oVPL202-203, and the Em^r^ gene expression cassette was amplified from pVPL2042 with oVPL1716-1717. pVPL3048 backbones, the Cm^r^ gene, and the Em^r^ gene expression cassette were assembled by LCR with bridging oligonucleotides (oVPL2414, -2415, -2529, and -2530) to yield pVPL3886. After transformation of LRΔФ2 with 5 μg pVPL3886, we confirmed single crossover with oligonucleotide combinations oVPL49-334-335 (upstream single crossover) and oVPL97-334-335 (downstream single crossover). Double-crossover recombinants were confirmed by PCR (oVPL334-335) and confirmation of Cm^s^ and Em^r^ phenotypes. The Em^r^ mutant of LRΔФ2 was named VPL4181 (LRΔФ2 Em^r^) (Fig. S1).

### Transmission electron microscopy.

Filtered cell-free phage suspensions (0.22-μm-pore PVDF filter) were resuspended in phage diluent (about 10^5^ PFU/ml). Five microliters of phage suspension was adsorbed onto a carbon-coated 200 mesh copper TEM grid (3.05 mm diameter [Gilder]). The phage particles were negatively stained with 2% (vol/vol) uranyl acetate staining solution and examined with a Tecnai T-12 electron microscope at an acceleration voltage of 120 kV and a magnification range of 20,000 to 200,000×.

### Phage production and survival of L. reuteri strains during murine GI transit.

Twenty 6-week-old male B6 mice (C57BL/6J) were purchased from Jackson Laboratory (Bar Harbor, ME). After arrival, animals were adjusted for 1 week to the new environment prior to the start of the experiment. Animals were housed at an environmentally controlled facility with a 12-h light and dark cycle. Food (standard chow [LabDiet, St. Louis, MO]) and water were provided *ad libitum*. Five groups (*n* = 4 per group) were gavaged for two consecutive days with a 100-μl phosphate-buffered saline (PBS) suspension containing 10^9^ CFU of rifampin-resistant derivatives of (i) the L. reuteri wild-type, (ii) the LRΔФ1 mutant, (iii) the LRΔФ2 mutant, (iv) the LRΔФ1 ΔФ2 mutant, and (v) the COMP strain. Fecal samples were collected from the bedding 16 h after the last oral administration and collected every following day after up to 6 days. Fecal content was resuspended in PBS to 100 mg/ml and plated on MRS agar plates containing 25 μg/ml rifampin. The fecal suspension was also used to determine the PFU/ml by a plaque assay as described above.

### *In vivo* colonization and competition in germfree mice.

Thirty-six germfree Swiss-Webster mice (12 to 16 weeks old) were maintained in sterile biocontainment cages in the gnotobiotic animal facility at the University of Alberta. Six treatment groups (*n* = 6 per group) were colonized following a single oral gavage of 100 μl L. reuteri cocktail in PBS (1:1 ratio, ∼10^9^ CFU). Each group was gavaged with a mixture of (i) WT Rif^r^ strain and sensitive host with Cm^r^, (ii) LRΔΦ1 Rif^r^ mutant and sensitive host with Cm^r^, (iii) LRΔΦ2 Rif^r^ and sensitive host with Cm^r^, (iv) LRΔΦ1 ΔΦ2 Rif^r^ mutant and sensitive host with Cm^r^, (v) complemented strain with Rif^r^ and sensitive host with Cm^r^, and (vi) LRΔΦ1 mutant with Cm^r^ and LRΔΦ2 mutant with Em^r^. Six days following colonization, fecal samples were collected from individual mice to determine the fecal CFU and PFU. At day 7, mice were sacrificed by CO_2_ asphyxiation and cecal contents and jejunal digesta were collected accordingly to determine the CFU and PFU per 100 mg content. To enumerate L. reuteri phages from intestinal contents, we performed the fecal plaque assay as described above. Mouse experiments were performed with approval of the Institutional Animal Care and Use Committee of the University of Alberta (Project ID 2099).

### Statistics.

Data representation was performed using DataGraph 4.3 (Visual Data Tools, Inc., Chapel Hill, NC). Statistical comparisons were performed using paired *t* test, one-way analysis of variance (ANOVA), and Tukey’s honestly significant difference (HSD) test (JMP Pro, version 11.0.0) for the multiple comparison between treatment groups in the animal experiment. Three biological replicates were performed for all *in vitro* studies. All samples were included in the analyses, and experiments were performed without blinding.

## Supplementary Material

Supplemental file 1
