# Supplementary material for: Prophages in Lactobacillus reuteri Are Associated with Fitness Trade-Offs but Can Increase Competitiveness in the Gut Ecosystem
Source: Appl Environ Microbiol. 2019 Dec 13;86(1):e01922-19. doi: 10.1128/AEM.01922-19 (PMC6912086; doi:10.1128/AEM.01922-19)
Supplement: Supplemental file 1 [file AEM.01922-19-s0001.pdf]

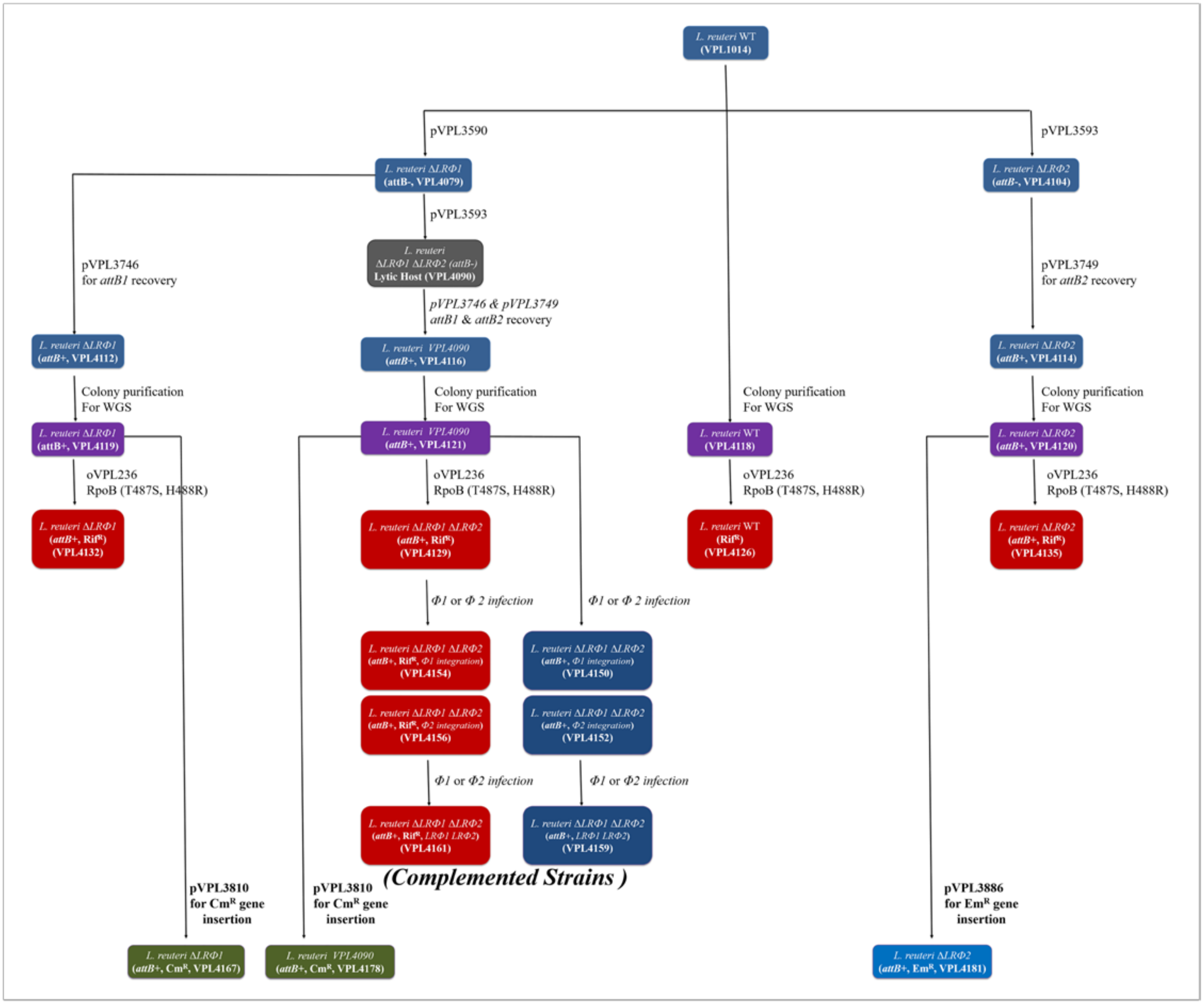

Figure S1. Schematic of *Lactobacillus reuteri* prophage-deletion derivatives constructed in this study. The schematic shows the consecutive construction of *L. reuteri* mutant derivatives listed from top to bottom.

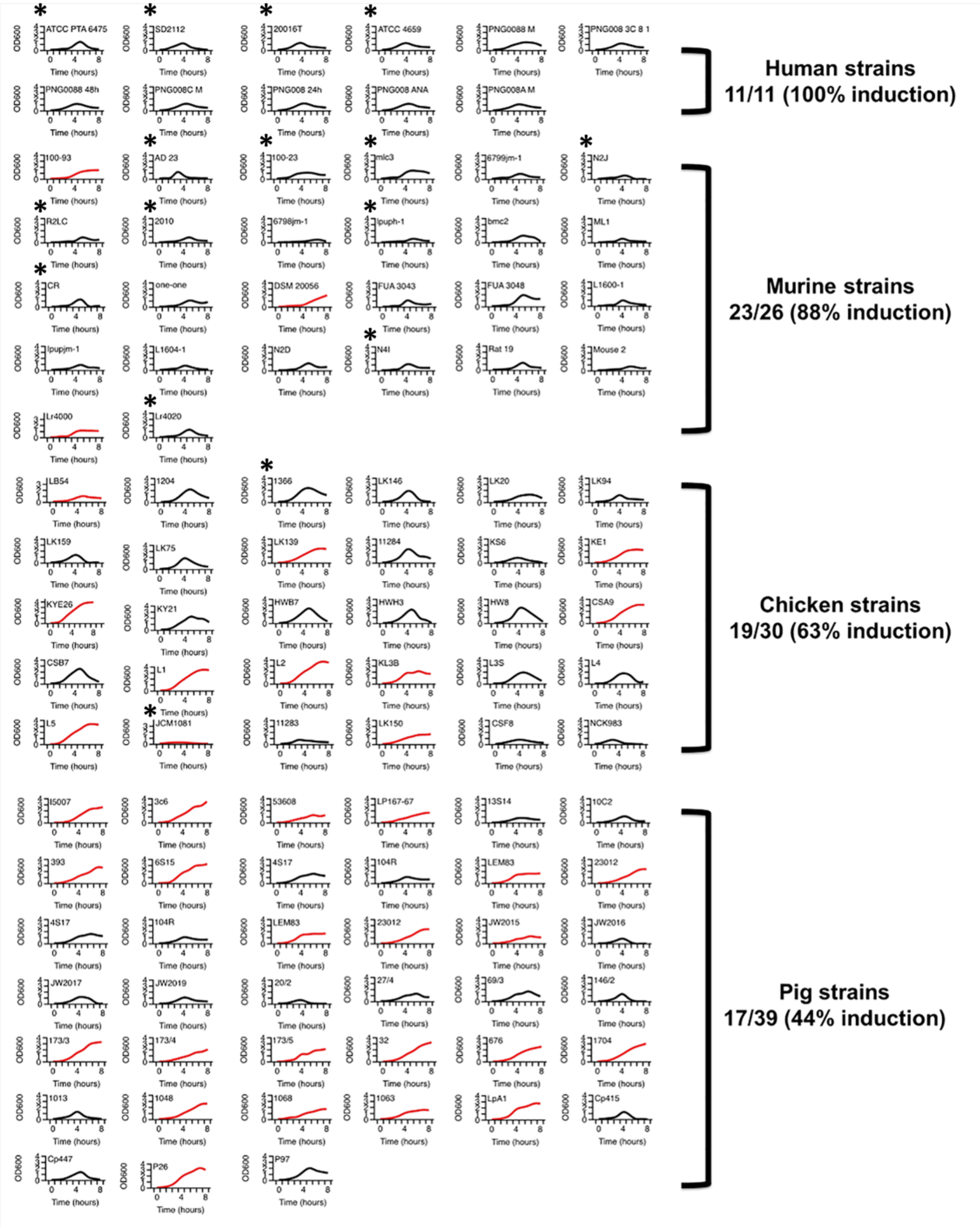

Figure S2. Growth of 106 *Lactobacillus reuteri* strains following mitomycin C induction. *L. reuteri* strains isolated from human (n=11), murine (n=26), chicken (n=30), and pig (n=39) were subjected to mitomycin C induction (0.5 µg/mL) during exponential growth (OD_{600} = 0.3) in MRS at 37°C, and growth was tracked for 8 hours. Growth curves in red represent growth patterns of strains that did not lyse following mitomycin C induction. Asterisks represent strains for which a genome sequence is available.

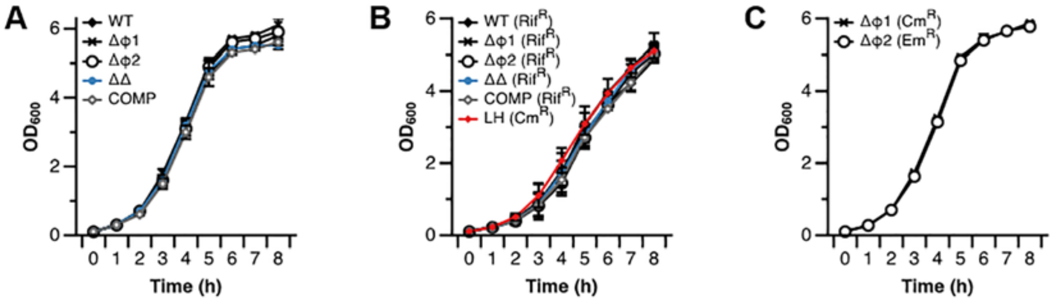

Figure S3. Growth comparison of *Lactobacillus reuteri* and its derivatives. (A) Optical cell density at 600 nm (OD_{600}; y-axis) over time (hours; x-axis) of *L. reuteri* wild-type (WT), *L. reuteri*ΔΦ1 (ΔΦ1), *L. reuteri*ΔΦ2 (ΔΦ2), *L. reuteri*ΔΦ1ΔΦ2 (ΔΔ), *L. reuteri*ΔΦ1ΔΦ2::Φ1::Φ2 (COMP). (B) Growth of rifampicin-resistant (Rif^R) derivatives of WT, ΔΦ1, ΔΦ2, ΔΔ, COMP and the chloramphenicol-resistant (Cm^R) derivative of the lytic host (LH). (C) Growth of the chloramphenicol-resistant (Cm^R) and erythromycin-resistant (Em^R) derivative of ΔΦ1 and ΔΦ2, respectively.
